# The TH1/TH2 Response Imbalance Zika Virus Pathogenesis: An Immunological Paradox

**DOI:** 10.1111/apm.70117

**Published:** 2025-12-17

**Authors:** Carla Menezes, Lívia Martins, Gustavo Ferro, Bernard Arnaud, Juarez Quaresma, Pedro Vasconcelos, Jorge Sousa

**Affiliations:** ^1^ Programa de Pós‐Graduação Em Biologia Parasitária da Amazônia Universidade Do Estado Do Pará Belém Brazil; ^2^ Seção de Arbovirologia e Febres Hemorrágicas Instituto Evandro Chagas Ananindeua Brazil; ^3^ Faculdade de Medicina Universidade Do Estado Do Pará Belém Brazil; ^4^ Departamento de Patologia Universidade Do Estado Do Pará Belém Brazil

**Keywords:** immunopathogenesis, inflammatory response, Th1/Th2 paradox, zika virus

## Abstract

Since its identification in 1947 and its global emergence in the Americas decades later, the Zika virus (ZIKV) has proven to be a multifaceted challenge to public health. Among the many aspects that remain poorly understood, the complex immunological paradox between Th1 and Th2 responses stands out, playing a central role in the immunopathogenesis of the infection. This narrative review critically explores how scientific literature has addressed this duality, highlighting that an exacerbated Th1 response—although effective in viral containment—is frequently associated with severe outcomes, such as heightened inflammation and autoimmune manifestations, including Guillain‐Barré syndrome. Conversely, a predominant Th2 profile, generally linked to milder clinical presentations, may favor viral persistence and, paradoxically, contribute to late‐onset neurological complications, such as encephalitis. Additionally, we discuss the central role of interactions between CD4 ^+^ and CD8^+^ T cells, monocytes, NK cells, and B cells, which, although essential for immune control of the infection, may, when dysregulated, exacerbate pathogenesis. By unraveling the mechanisms behind this delicate balance between antiviral defense and immune‐mediated damage, we aim to shed light on new therapeutic perspectives. Understanding the nature and consequences of this Th1/Th2 paradox is crucial for guiding the development of immunomodulatory strategies that foster an effective response against ZIKV, while minimizing the risk of neurological injury.

## 1 Introduction

### 1.1 ZIKV and Its Global Impact

ZIKV, first identified in 1947, spread globally and reached the Americas in 2015, where a significant increase in microcephaly cases and an association with Guillain‐Barré syndrome (GBS) were observed. Local transmission of the virus was rapidly detected, prompting a public health alert owing to its serious neonatal and neurological consequences. ZIKV, a member of the *Flaviviridae* family, interacts with the human immune system and triggers complex responses, including activation of T cells and the release of inflammatory cytokines, which may contribute to the complications associated with infection. This situation led to a health emergency that lasted 10 months, ending when the World Health Organization (WHO) declared its conclusion [1, 2, 3, 4, 5].

ZIKV infection has raised public health concerns not only due to its rapid transmission and serious neurological outcomes, but also due to increasing interest in its interaction with the human immune system [6, 7, 8, 9]. Studies have shown that ZIKV can modulate immune responses, particularly by evading or manipulating both innate and adaptive defense mechanisms [10, 11, 12]. These immune interactions may underlie the severity of clinical manifestations and challenges in controlling infection, highlighting the paradoxical nature of the host response. Although the Th1 immune response is crucial for fighting the virus, it can also trigger exacerbated inflammation that aggravates neurological complications such as GBS. On the other hand, a Th2 profile, which tends to be less inflammatory, may inadvertently facilitate viral persistence, leading to other neurological complications such as encephalitis. Understanding these specific immune components is therefore vital for the development of strategies that balance combating the virus with mitigating damage to the host [13, 14, 15, 16].

However, studies exploring this immune dynamic remain limited, particularly in Brazil, where ZIKV has had a significant impact on public health. This gap highlights the importance of further in‐depth studies to advance scientific understanding and inform the development of targeted and personalized immunomodulatory interventions, especially for vulnerable populations. Accordingly, this narrative review aims to examine the scientific literature on the Th1/Th2 paradox in the immunopathogenesis of ZIKV infection [17, 18, 19, 20, 21].

### 1.2 Immune System and Response to ZIKV

The immune system comprises two primary components: innate and adaptive immunity. Innate immunity is inherited, present from birth, and characterized by a rapid, nonspecific response [22, 23]. It includes physical, chemical, and biological barriers such as the skin and the digestive, respiratory, and genitourinary systems, as well as phagocytic cells, such as neutrophils, monocytes, and macrophages, and complement system proteins, which are activated upon contact with pathogens. In addition, the innate immune system includes natural killer (NK) cells, which play an essential role in eliminating infected or abnormal cells. Although initially effective, innate immunity does not confer immunological memory [24, 25, 26, 27, 28].

In contrast, adaptive immunity is specific and possesses memory, allowing for faster and more robust responses upon reexposure to the same pathogen. It involves B lymphocytes (or B cells), which are responsible for humoral immunity via antibody production, and T lymphocytes (or T cells), which mediate cellular immunity. Primary lymphoid organs, such as the thymus and bone marrow, and secondary organs, such as the lymph nodes, tonsils, and spleen, are essential for the maturation and storage of these immune cells [29, 30, 31].

During viral infections, the immune system is activated by cytokines and interferons (IFNs), which initiate the destruction of infected cells and the production of antibodies. In ZIKV infection specifically, the roles of the T helper 1 (Th1) and T helper 2 (Th2) responses, subsets of T helper cells that modulate immunity, are particularly significant [32, 33].

Maintaining a balance between Th1 and Th2 responses is critical for preventing severe complications. In ZIKV infection, as mentioned in the introduction, an exacerbated Th1 response has been linked to conditions such as GBS, whereas Th2 predominance in pregnant women may contribute to fetal complications, including microcephaly. These immune responses are modulated by both viral factors, such as ZIKV proteins that direct immune polarization, and host factors, including genetic predisposition and preexisting immunity. In the next section, the Th1/Th2 paradox and its clinical implications will be discussed [34, 35, 36].

### 1.3 TH1/TH2 Immune Paradox: Concept and Relevance in ZIKV Infection

Zika virus infection presents a unique immunological scenario defined by the Th1/Th2 paradox. In both experimental and clinical settings, a predominant Th1 response has been observed, characterized by IFN‐γ, TNF‐α, and IL‐2 production, alongside activation of cytotoxic CD4^+^ and CD8^+^ T cells. While this profile supports viral clearance, it also contributes to inflammatory damage, particularly in the central nervous system [37]. Conversely, fatal cases of congenital Zika syndrome (CZS) reveal simultaneous expression of Th2‐related cytokines (IL‐4, IL‐10, TGF‐β) and mediators of Th9, Th17, and Treg pathways, indicating that regulatory mechanisms act in parallel to Th1‐driven antiviral responses to limit excessive inflammation [37]. This coexistence of opposing signals defines the Th1/Th2 paradox in ZIKV infection.

Innate immune activation begins with the recognition of viral PAMPs by TLRs and RLRs, especially TLR3, whose activation induces IL‐6 production and STAT3–SOCS3 signaling, suppressing STAT1 phosphorylation and type I IFN responses [38, 39, 40, 41, 42, 43]. This inhibition favors viral replication, leading to sustained inflammation and a shift toward Th1 polarization with IFN‐γ and TNF‐α secretion, potentially worsening neuroinflammatory injury [42, 43, 44, 45]. Hence, TLR3 signaling modulates both antiviral and inflammatory pathways, altering the Th1/Th2 balance and contributing to ZIKV neuropathogenesis.

Studies of ZIKV‐infected pregnant women show expansion of Th1 and Th2 memory cells in mothers but predominance of naïve T cells in neonates [7]. This suggests age‐ and exposure‐dependent immune plasticity and demonstrates that Th1 and Th2 components operate concurrently, modulating disease outcomes. Histopathological findings in neonates with CZS show lymphocyte and macrophage infiltrates expressing IFN‐γ, TNF‐α, and IL‐2—hallmarks of Th1 activation—coexisting with IL‐10 and TGF‐β, reflecting partial regulatory control [37]. Accumulation of IL‐17, IL‐22, and IL‐23‐positive cells and chemokines such as RANTES and IP‐10 indicates robust Th17 axis activation and continuous effector recruitment, amplifying both antiviral defense and tissue damage [37].

At the maternal–fetal interface, placental tissues exhibit elevated IL‐1β, IL‐6, and TGF‐β, fostering Th17 differentiation within a tolerogenic environment. The coexistence of inflammatory and regulatory mediators underscores the immunological tension between antiviral defense and fetal tolerance. T cell and macrophage infiltration in the intervillous space highlights viral disruption of maternal–fetal immune homeostasis [7]. Dysregulation of the iNOS/arginase axis and altered VEGF and FGF expression suggest additional vascular and neurodevelopmental consequences [37].

Experimental models further demonstrate that ZIKV induces Th1‐like T follicular helper (Tfh) cells that produce IFN‐γ and support long‐lasting IgG2c responses, linking Th1 transcriptional programs (T‐bet/IFN‐γ) to humoral immunity [46]. Although protective, these Th1‐like Tfh responses may exacerbate local inflammation in the fetal CNS and placenta, especially in tolerance‐dependent contexts such as pregnancy [7, 46]. Regulation of type I IFN signaling shapes the magnitude and quality of these responses, influencing the balance between antiviral control and immunopathology [46].

During the acute phase of infection, a transient increase in multiple cytokine profiles (Th1, Th2, Th9, and Th17) occurs, followed by chemokine predominance (IP‐10, RANTES) during recovery [47]. Unlike dengue, IFN‐γ and TNF‐α show limited elevation, indicating partial Th2 skewing. A Th1‐dominant response accelerates viral clearance but enhances immunopathology, while excessive Th2 activity suppresses Th1 mechanisms, enabling viral persistence [48, 49, 50, 51, 52, 53, 54]. Elevated IL‐4 and IL‐10 levels correlate with milder disease yet prolonged infection [55, 56]. Conversely, dysregulated Th1 activity has been linked to complications such as microcephaly and Guillain–Barré syndrome [37, 47, 57, 58, 59, 60, 61, 62].

**FIGURE 1:**
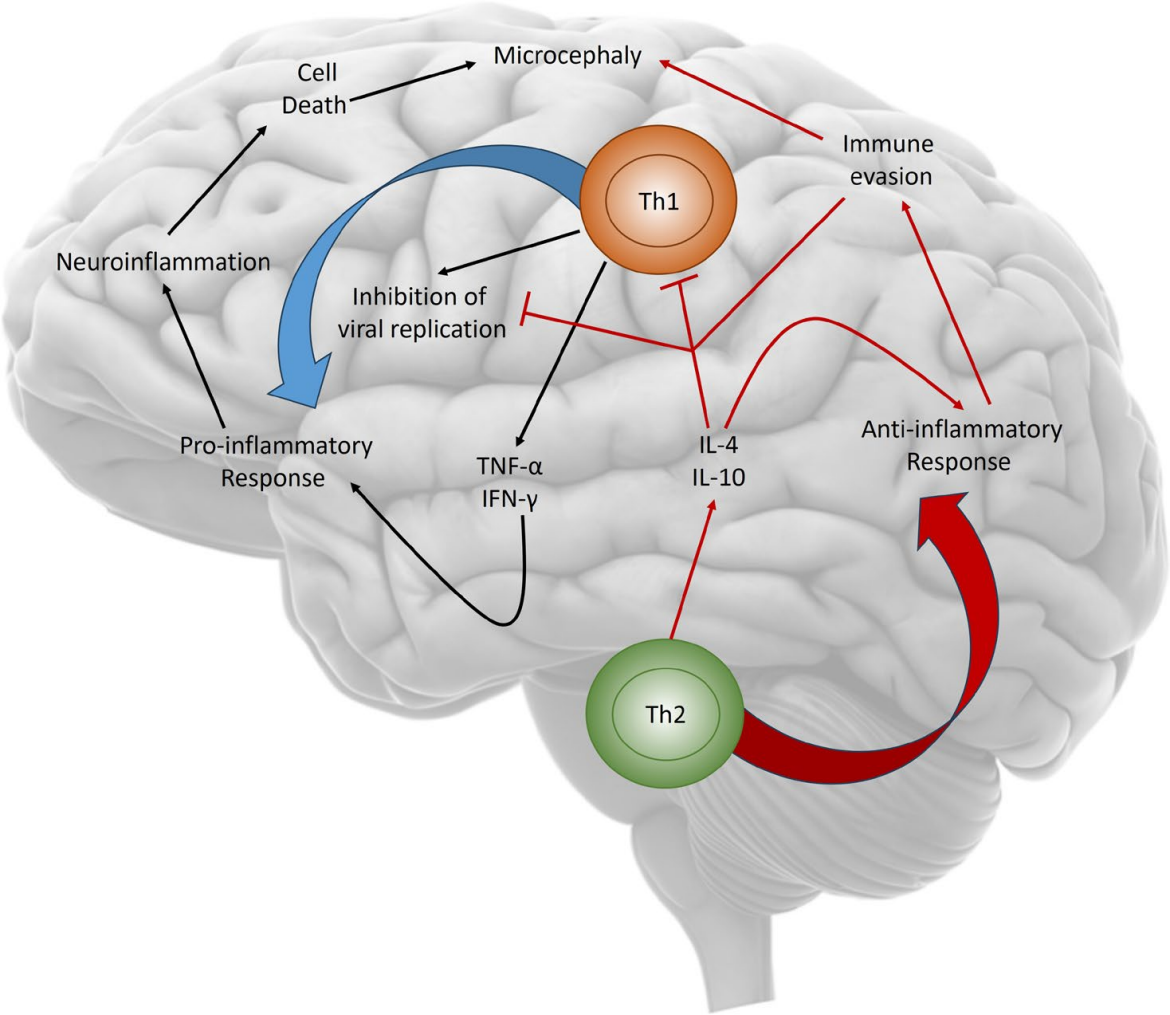
Th1/Th2 immune paradox during ZIKV infection. This figure illustrates the immunological paradox between Th1 and Th2 responses during ZIKV infection. The Th1 response, characterized as pro‐inflammatory and primarily mediated by TNF‐α and IFN‐γ, plays a crucial role in inhibiting viral replication. However, excessive activation of this pathway may lead to neuroinflammation and neuronal cell death, contributing to neurological complications such as microcephaly. Conversely, the Th2 response is anti‐inflammatory and typically associated with cytokines such as IL‐4 and IL‐10, promoting the regulation and resolution of inflammation. Predominance of the Th2 response, however, may suppress Th1‐mediated antiviral activity, enabling immune evasion and facilitating viral persistence, factors that may also be linked to microcephaly and other neurological complications associated with ZIKV infection.

Exaggerated Th2 polarization may facilitate antibody‐dependent enhancement (ADE) through cross‐reactive antibodies with other flaviviruses, promoting viral entry into macrophages and amplifying inflammation [63, 64, 65, 66, 67, 68]. Thus, ZIKV immunopathogenesis reflects a dynamic balance between pro‐inflammatory and regulatory axes. Protective Th1 and humoral Th2/Tfh responses coexist within microenvironments—such as the CNS and placenta—where immune regulation is essential, turning antiviral defense into potential immunopathology risk [7, 46, 69, 70, 71]. Figure 1 illustrates these complex interactions that underline the immunological paradox between Th1 and Th2 responses during ZIKV infection.

Therefore, an imbalance in Th1/Th2 responses carries significant implications for the neuropathogenesis of ZIKV infection. A deeper understanding of these immunological mechanisms is essential for developing more effective therapeutic strategies to prevent and treat ZIKV infections and associated neurological complications [72].

### 1.4 Mechanisms Underlying the TH1/TH2 Paradox in ZIKV Infection

The coordinated action of CD4^+^ and CD8^+^ T cells, monocytes, NK cells, and neutralizing antibodies is essential for effective ZIKV control but can also drive immunopathological outcomes. Proper regulation of these components maintains antiviral efficiency, whereas an imbalance in Th1/Th2 signaling amplifies inflammatory injury and contributes to complications such as GBS and microcephaly [73, 74, 75].

CD4^+^ T cells orchestrate immune activation and differentiation, polarizing into Th1 or Th2 subsets with distinct consequences. Th1 responses, dominated by IFN‐γ, TNF‐α, and IL‐2, promote cytotoxic CD8^+^ and NK cell activity for viral clearance. In contrast, Th2 responses, characterized by IL‐4 and IL‐13, shift immunity toward antibody production and tissue repair while limiting inflammation. The balance between these axes defines the nature and magnitude of both innate and adaptive responses [76, 77, 78]. As summarized in Table 1, Th1 and Th2 profiles differ in their cytokines, effector functions, and implications for disease progression, which are critical for understanding the immune dynamics during ZIKV infection.

**TABLE 1:** Comparison between T helper 1 (Th1) and T helper 2 (Th2) cell‐associated immune responses and their implications in Zika virus (ZIKV) infection.

Aspect	Th1 response	Th2 response	Clinical implications	References
Types of response	Cellular immunity (cytotoxic, pro‐inflammatory).	Humoral immunity (antibody‐mediated, anti‐inflammatory).	Exacerbated Th1 response is associated with an increased risk of fetal neural damage and Guillain Barré syndrome (GBS).	[62]
Main cells	CD4+ Th1 lymphocytes, CD8+ T lymphocytes, macrophages, and natural killer (NK) cells.	CD4+ Th2 lymphocytes and B lymphocytes.	Th1 lymphocyte infiltratione in the central nervous system (CNS) is associated with neuroinflammation in both adults and fetuses.	[60]
Cytokines involved	Interferon‐gamma (IFN‐γ), tumor necrosis factor‐alpha (TNF‐α), and interleukin‐2 (IL‐2).	IL‐4, IL‐5, IL‐10, and IL‐13.	Elevated levels of IFN‐γ and TNF‐α are associated with fetal growth restriction, microcephaly, and placentates.	[34]
Role of ZIKV infection	Enhances viral elimination, but increases the risk of tissue damage.	Promotes immune tolerance and reduces inflammation.	Th1 activation lowers viral load but exacerbates tissue damage in the CNS and placenta.	[79]
Pregnancy paradox	Th1 activation compromises placental immunotolerance and may cause miscarriage or malformations.	Th2 responses preserves pregnancy but impairs infection control.	A pro‐Th1 imbalance during pregnancy is strongly linked to congenital Zika syndrome.	[53]
Associated complications	GBS, encephalitis, fetal microcephaly, intrauterine growth restriction.	Asymptomatic or persistent infection, risk of prolonged viremia.	Th1 responses favor severe neurological complications in adults and fetuses Th2 responses may mask chronic infection.	[14]
Clinical outcomes	Rapid viral control with a high risk of permanent neurological sequelae.	Increased risk of persistent infection, but reduced fetal immune‐mediated damage.	Clinical severity correlates with the intensity of Th1 response and its associated neurotoxicity.	[78]

Monocytes and their derivatives—macrophages and dendritic cells—serve as key intermediates between innate and adaptive immunity. Under Th1 influence, IFN‐γ enhances their phagocytic and antigen‐presenting capacities, supporting T and NK cell activation. However, excessive stimulation may trigger hyperinflammation, contributing to GBS‐like pathology [80, 81, 82]. Conversely, Th2 cytokines such as IL‐6 and IL‐10 promote M2 macrophage polarization and regulatory activity, favoring tissue recovery but potentially compromising viral clearance [46, 82].

NK cells are pivotal effectors activated downstream of Th1 cytokines, eliminating infected cells through perforin‐ and granzyme‐mediated cytotoxicity. Yet, their uncontrolled activation sustains the inflammatory loop that underlies ZIKV‐associated neuropathology [80, 81, 82].

Humoral immunity represents another layer of this network. Neutralizing antibodies block viral entry and mediate opsonization, but their quality depends on T helper regulation. IFN‐γ from Th1 cells promotes class switching toward IgG subclasses with enhanced complement and Fc receptor binding, strengthening antiviral efficacy [83, 84]. Meanwhile, Th2 cytokines such as IL‐4 drive B cell activation and antibody secretion, ensuring viral neutralization. When dysregulated, however, low‐affinity antibodies and immune complex formation can intensify inflammation, linking Th1/Th2 imbalance to neurological complications such as microcephaly [85, 86].

**FIGURE 2:**
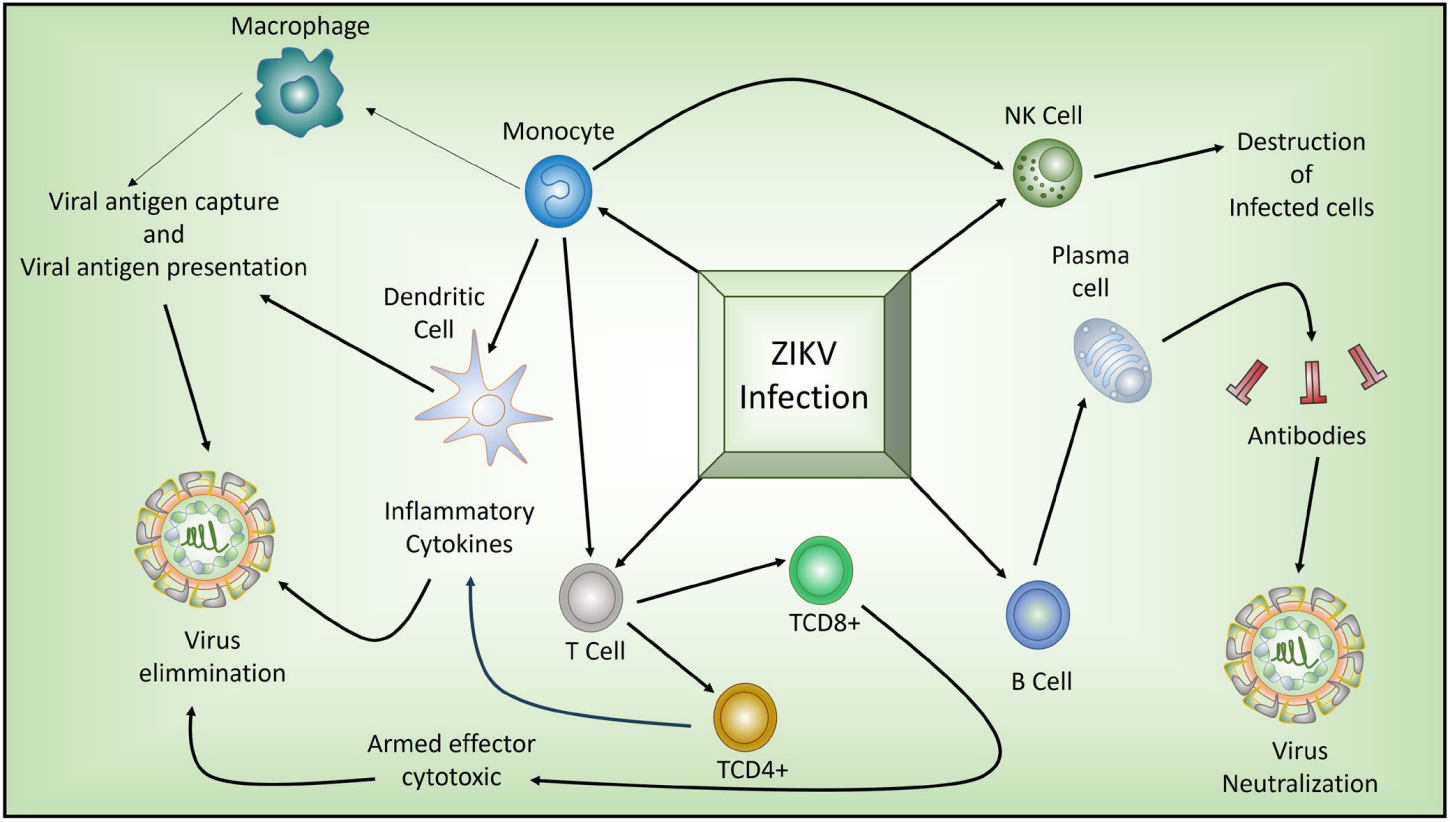
Immune cell responses to ZIKV infection. The figure illustrates the key immune responses in the human body during ZIKV infection, involving four primary cell groups: T cells, monocytes, NK cells, and B cells. T cells are subdivided into CD4+ and CD8+ subsets, each with distinct functional characteristics. CD4+ T cells facilitate the immune response by promoting the production of inflammatory cytokines, such as IFN‐γ, which are essential for viral clearance. CD8+ cells act as cytotoxic effectors, targeting and destroying virus‐infected cells. Monocytes differentiate into macrophages and dendritic cells, both of which are involved in antigen capture and presentation, initiating adaptive immune responses necessary for viral clearance. NK cells, similar to CD8+ cells, are also capable of destroying infected cells. B cells can differentiate into plasma cells and mediate humoral immune responses, producing neutralizing antibodies that block virus replication and facilitate its elimination.

In sum, the Th1/Th2 paradox in ZIKV infection emerges from the need to balance effective antiviral responses with immune regulation. Excessive Th1 activity promotes cytotoxic damage, whereas Th2 dominance impairs viral clearance and may predispose to antibody‐mediated enhancement. The clinical spectrum of ZIKV, therefore, reflects the outcome of this dynamic equilibrium. Figure 2 illustrates the immune responses of various cell types involved in the defense against ZIKV infection.

The dynamic interaction among various immune cells, depending on the type and intensity of the response, can lead to either effective viral control or severe complications. Therefore, a well‐regulated interplay among T cells, monocytes, NK cells, and neutralizing antibodies is crucial for controlling ZIKV infection. Understanding how these cells interact and their regulatory mechanisms is crucial for developing more effective and targeted therapeutic strategies against ZIKV infection [87, 88, 89].

### 1.5 Immunity, Cross‐Reactivity and Vaccination in the Context of the TH1/TH2 Paradox

Cross‐reactive antibodies and autoantibody production are central to ZIKV immunopathogenesis. Prior immunity to dengue virus (DENV) can generate cross‐reactive antibodies that, instead of providing full protection, may enhance ZIKV infection via antibody‐dependent enhancement (ADE). Subneutralizing antibodies facilitate viral entry into Fc receptor‐bearing cells, particularly monocytes and macrophages, increasing viral replication and systemic inflammation [90]. Th2‐skewed responses amplify this effect by promoting excessive antibody production, whereas insufficient Th1 activity limits viral clearance, highlighting the role of the Th1/Th2 paradox in disease severity [91].

Autoantibodies induced during ZIKV infection contribute to autoimmune complications such as GBS. Molecular mimicry between viral antigens and host neural components triggers immune‐mediated peripheral nerve damage, a process intensified by preexisting cross‐reactive antibodies from prior DENV exposure [92, 93, 94, 95]. Here, Th2 responses favor autoantibody production, while Th1‐driven inflammation exacerbates neural injury, illustrating how Th1/Th2 imbalance drives both antiviral and autoimmune pathology.

Viral antigen structure shapes immune polarization and vaccine responses. The NS5 protein, essential for replication and immune evasion, influences host signaling pathways that bias Th responses, while ZIKV Domain III (zDIII) is a key target for neutralizing antibodies and vaccine design [96]. DNA vaccine trials have shown that Th1‐biased responses promote effective cellular immunity and long‐lasting antibody production, demonstrating the importance of Th1 polarization in protective immunity [97].

Cross‐reactivity among flaviviruses further complicates immunity. Structural similarity in zDIII generates antibodies that may recognize both ZIKV and DENV. While partially protective, these antibodies can enhance infection via ADE, particularly under Th2‐biased conditions that favor humoral over cellular antiviral responses. Conversely, NS5 inhibits interferon signaling but represents a stable therapeutic target among flaviviruses [98, 99].

These dynamics underscore the impact of the Th1/Th2 paradox on vaccination strategies. Th1 polarization is desirable to ensure durable cellular and humoral immunity, whereas excessive Th2 bias increases the risk of low‐affinity antibody production, cross‐reactivity, and ADE. Vaccine development in regions with co‐circulating flaviviruses must therefore balance Th1/Th2 responses to maximize protection while minimizing immunopathological risks [100, 101, 102, 103, 104].

### 1.6 Challenges and Future Prospects

A major challenge is the cross‐reactivity between ZIKV and DENV. The presence of antibodies against DENV can facilitate the entry of ZIKV into host cells via ADE, thereby worsening the infection [101]. Additionally, the presence of autoantibodies has been observed and may contribute to autoimmune diseases associated with ZIKV infection. The NS5 and zDIII proteins have emerged as promising immunodominant antigens for the development of vaccines and diagnostic tools. Technologies such as VLPs are safe and effective alternatives. However, the structural similarity among flaviviruses poses challenges for achieving vaccine specificity. This cross‐reactivity and the development of autoantibodies are directly influenced by the Th1/Th2 immune paradox, as excessive Th2 responses favor low‐affinity or cross‐reactive antibodies while insufficient Th1 activity reduces cellular antiviral control. Future immunological strategies should consider the Th1/Th2 immune paradox and the epidemiological context in endemic regions [105, 106, 107, 108].

## 2 Conclusion

This review highlights the Th1/Th2 immunological paradox as a central feature of ZIKV pathogenesis. The balance between Th1 and Th2 responses determines both the efficiency of viral clearance and the severity of immunopathology. Exacerbated Th1 responses, while crucial for antiviral defense, can drive collateral tissue damage, including Guillain–Barré syndrome, through excessive pro‐inflammatory cytokine release. Conversely, Th2 dominance may reduce inflammation but favor viral persistence, contributing to complications such as encephalitis and prolonged infection.

Immune cells—including CD4^+^ and CD8^+^ T lymphocytes, monocytes, and NK cells—oscillate between protective and pathological roles depending on the prevailing immune profile, exemplifying the paradox where protective responses may simultaneously cause harm. Understanding this balance is essential for developing therapeutic and preventive strategies, particularly in the context of cross‐reactivity among flaviviruses and vaccine‐induced immune responses. Future research should further explore the interactions between immune components and cellular changes during ZIKV infection to inform interventions that minimize immunopathology while maintaining effective antiviral immunity.

## Funding

This work was supported by the National Council for Scientific and Technological Development (CNPq) through the National Institute of Science and Technology (INCT) – INCT‐VER project (grant No. 406360/2022‐7), and by the Coordenação de Aperfeiçoamento de Pessoal de Nível Superior – Brasil (CAPES).

## Consent

The authors have nothing to report.

## Conflicts of Interest

The authors declare no conflicts of interest.

## Data Availability

Research data are not shared.
